# miR-200a-3p in Human Umbilical Cord Mesenchymal Stem Cell-Derived Exosomes Attenuates UVB-Induced Skin Inflammatory Response and Oxidative Stress via Keap1-Nrf2 Pathway

**DOI:** 10.1155/sci/7831890

**Published:** 2025-10-28

**Authors:** Qixiang Gui, Neng Ding, Jinyue Liu, Yunpeng Zhao, Antong Du, Jie Zhu, Haimei Wu, Minjuan Wu, Yue Wang, Lie Zhu

**Affiliations:** ^1^Department of Plastic and Reconstructive Surgery, Second Affiliated Hospital of Naval Medical University (Shanghai Changzheng Hospital), Shanghai 200003, China; ^2^Department of Plastic and Reconstructive Surgery, Shanghai East Hospital, School of Medicine, Tongji University, Shanghai 200120, China; ^3^Department of Burns and Plastic Surgery, PLA 74th Group Army Hospital, Guangzhou 510300, China; ^4^Department of Stem Cell and Regeneration Medicine, Translational Medicine Research Center, Naval Medical University, Shanghai 200433, China; ^5^Faculty of Health Sciences and Engineering, University of Shanghai for Science and Technology, Shanghai 200093, China; ^6^Department of Histology and Embryology, Basic Medicine Collage, Naval Medical University, Shanghai 200433, China; ^7^Department of Stem Cell Engineering, Institute of Stem Cell Research and Clinical Translation, Shanghai 200120, China; ^8^Department of Stem Cell Engineering, Shanghai Key Laboratory of Cell Engineering, Shanghai 200433, China

**Keywords:** exosomes, inflammatory response, Keap1-Nrf2 pathway, miR-200a-3p, oxidative stress, UVB

## Abstract

Ultraviolet (UV) radiation induces skin damage primarily through oxidative stress and excessive inflammation. Exosomes derived from mesenchymal stem cells have emerged as promising therapeutic agents for tissue repair. Here, we investigated the protective effects of human umbilical cord mesenchymal stem cell-derived exosomes (HuMSC-Exos) on UVB-induced skin injury in HaCaTs and C57BL/6 mice. HuMSC-Exos significantly reduced reactive oxygen species (ROS) levels, suppressed proinflammatory cytokines (IL-1β, TNF-α, and IL-6), and improved cell migration. Mechanistically, HuMSC-Exos inhibited Keap1, enhanced both total and phosphorylated Nrf2 expression, promoted its nuclear translocation, and upregulated antioxidant genes (*HMOX1*, *NQO1*, *CAT*, and *SOD2*). miR-200a-3p in HuMSC-Exos mediated these effects by targeting Keap1. Furthermore, preliminary data suggested that HuMSC-Exos also attenuate inflammatory responses via the NF-κB pathway. In vivo, HuMSC-Exos attenuated UVB-induced skin injury and inflammation by activating the Nrf2 signaling cascade. Collectively, our findings reveal a novel protective mechanism and highlight the therapeutic potential of HuMSC-Exos in mitigating UV-induced skin damage by modulating oxidative stress and inflammation.

## 1. Introduction

Solar ultraviolet (UV) radiation is a major environmental factor implicated in the onset and progression of various skin disorders, including chronic actinic dermatitis, polymorphic light eruption, photoaging, and photocarcinogenesis [1]. Based on wavelength, UV radiation is classified into UVA (320–400 nm), UVB (280–320 nm), and UVC (200–280 nm) [2]. Among these, UVB radiation exerts the most deleterious effects on the epidermis due to its limited tissue penetration, primarily affecting keratinocytes (HaCaTs), the predominant epidermal cells responsible for maintaining skin homeostasis and barrier function [3]. UVB exposure triggers a cascade of pathophysiological events in keratinocytes, including excessive reactive oxygen species (ROS) generation, DNA damage, gene mutations, and inflammatory activation [4]. Notably, oxidative stress and inflammation interact in a vicious cycle: ROS overproduction activates proinflammatory signaling pathways, which, in turn, exacerbate oxidative stress, thereby amplifying tissue damage [5]. Consequently, targeting both oxidative stress and inflammatory responses in keratinocytes represents a rational strategy for mitigating UVB-induced skin injury.

UVB radiation has been shown to upregulate key inflammatory mediators such as cyclooxygenase-2 (COX-2; encoded by *PTGS2*) and inducible nitric oxide synthase (iNOS; encoded by *NOS2*), both of which play pivotal roles in inflammatory cascades [6]. COX-2 catalyzes the conversion of arachidonic acid to prostaglandins such as PGE2, potent mediators of inflammation [7], while iNOS generates nitric oxide, a critical signaling molecule involved in immune and inflammatory processes [8]. In addition, UVB stimulates keratinocytes to secrete proinflammatory cytokines including TNF-α, IL-1β, and IL-6 [9], further amplifying cutaneous inflammatory responses and tissue damage.

The nuclear factor erythroid 2-related factor 2 (Nrf2; encoded by *NFE2L2*) signaling pathway serves as a central defense mechanism against oxidative stress. Under basal conditions, Nrf2 is sequestered in the cytoplasm by its negative regulator Kelch-like ECH-associated protein 1 (Keap1; encoded by *KEAP1*), which mediates Nrf2 ubiquitination and proteasomal degradation. Oxidative stress disrupts the Keap1–Nrf2 interaction by modifying critical cysteine residues on Keap1, thereby stabilizing Nrf2 and facilitating its nuclear translocation. In the nucleus, Nrf2 forms a heterodimer with small Maf proteins and binds to antioxidant response elements (AREs) to induce the expression of cytoprotective genes, such as NAD(P)H quinone oxidoreductase-1 (NQO1; encoded by *NQO1*) and heme oxygenase-1 (HO-1; encoded by *HMOX1*) [10]. Beyond its antioxidant function, Nrf2 also exerts anti-inflammatory effects, underscoring its potential as a therapeutic target in UVB-induced skin damage [11].

Human umbilical cord mesenchymal stem cells (HuMSCs) have emerged as a promising source for regenerative medicine owing to their easy accessibility, low immunogenicity, and robust paracrine activity [12, 13]. Increasing evidence suggests that MSCs exert their therapeutic effects largely via exosomes, nanosized extracellular vesicles enriched with bioactive molecules such as proteins, lipids, and nucleic acids. For instance, exosomes derived from HuMSCs (HuMSC-Exos) enriched in miR-21-5p and let-7b-5p have been shown to promote hair shaft regrowth via the Wnt/β-catenin pathway [14], while MSC-Exos have demonstrated efficacy in alleviating UVB-induced photoaging [15]. Among exosomal cargos, microRNAs (miRNAs) play critical roles in posttranscriptional regulation by transferring into recipient cells and modulating key signaling pathways. Notably, several studies have reported that miR-200a directly targets Keap1, thereby activating Nrf2 signaling and enhancing antioxidant responses in various pathological contexts [16, 17]. However, the potential role of exosomal miR-200a in mitigating UVB-induced oxidative stress and inflammation in skin remains largely unexplored.

Therefore, this study aimed to investigate the antioxidant and anti-inflammatory effects of HuMSC-Exos on UVB-irradiated HaCaTs and elucidate the involvement of the Keap1–Nrf2 pathway. We employed RNA sequencing and bioinformatics analyses to identify miRNAs potentially mediating these effects and validated the role of miR-200a-3p in regulating Keap1–Nrf2 signaling. Finally, the therapeutic efficacy of HuMSC-Exos was further assessed in UVB-irradiated C57BL/6 mouse models. Collectively, our findings provide novel mechanistic insights into the protective role of HuMSC-Exos against UVB-induced skin injury and highlight their potential as a cell-free therapeutic approach for photoprotection.

## 2. Methods

### 2.1. Cell Culture

HuMSCs were obtained from the Department of Histology and Embryology at the Naval Medical University [18]. Cells were maintained at 37°C in a humidified atmosphere containing 5% CO_2_ using mesenchymal stem cell basal medium (Cell Farm, Shanghai, China) supplemented with 10% exosome-free fetal bovine serum (FBS; catalog number 10099141, Gibco, USA). HuMSCs at passages 2–6 were used for all experiments, and culture supernatants were collected for subsequent exosome isolation.

HaCaTs were procured from Cobioer Biosciences (Nanjing, China) and cultured in high-glucose Dulbecco's modified eagle medium (DMEM; Gibco, USA) supplemented with 10% FBS and 1% penicillin-streptomycin (Sigma, USA). Experiments were performed using cells between passages 8 and 12.

### 2.2. Isolation and Identification of Exosomes

Exosomes were isolated from HuMSCs using differential centrifugation methods [19]. Briefly, conditioned medium was collected and kept at 4°C throughout the procedure. The medium was sequentially centrifuged at 300 × *g* for 10 min to remove cells, 2000 × *g* for 15 min to eliminate cell debris, and 16,500 × *g* for 20 min to discard large vesicles and residual impurities. The resulting supernatant was filtered through a 0.22 μm membrane to remove potential microbial contaminants and subsequently ultracentrifuged at 100,000 × *g* for 60 min to pellet exosomes. The exosome pellets were washed with 1–2 mL of PBS and subjected to a second ultracentrifugation at 100,000 × *g* for 60 min to further enhance purity. Finally, purified exosomes were resuspended in an appropriate volume of PBS and stored at −80°C until use.

Exosome morphology was examined using transmission electron microscopy (JEM-2100 TEM, JEOL, Japan). Particle size distribution and concentration were assessed by nanoparticle tracking analysis (NTA, NanoSight LM10, Malvern Instruments, UK). Exosomal surface markers were confirmed by western blotting. To visualize cellular uptake, HuMSC-Exos were labeled with DiO (Beyotime, China) and incubated with HaCaTs for 12 h, whose nuclei were stained with Hoechst 33342 (Beyotime, China). Uptake was then observed using a laser scanning confocal microscope (LSM900, Carl Zeiss, Germany).

### 2.3. Cell Counting Kit-8

Cell viability assays were performed to determine the optimal concentration of HuMSC-Exos for subsequent experiments. HaCaTs were incubated with increasing concentrations of HuMSC-Exos (0, 6.25, 12.5, 25, 50, and 100 μg/mL) for 24 h prior to UVB irradiation. Cell viability was then assessed using the cell counting kit-8 (CCK-8; Beyotime, China), following established protocols [20, 21]. Briefly, after incubation, the culture medium was replaced with 10 μL of CCK-8 solution and 90 μL of serum-free medium per well. Plates were incubated for an additional 2 h at 37 °C, after which the absorbance at 450 nm was measured using a microplate reader (Tecan, Thermo Scientific, USA). Cell viability (%) was calculated as follows: cell viability (%) = (sample−blank)/(control−blank) × 100%.

### 2.4. UVB Induced Damage of HaCaTs and HuMSC-Exos Pretreatment

UVB-induced cellular injury was established as previously described [22]. Briefly, HaCaTs were seeded in 6-well plates and, following removal of the culture medium and rinsing with PBS, were exposed to UVB irradiation using a UV cross-linker (model PL-L36W/01/4P, Philips, Poland) with a peak emission at 312 nm (UVB range: 280–320 nm). Cells were irradiated at doses ranging from 15 to 200 mJ/cm^2^, with exposure time adjusted to achieve the desired dose. A UV meter (Solar Light Co., USA) was used to ensure accurate dosage measurement. After irradiation, fresh medium was added, and cells were incubated under standard culture conditions for 24 h [23]. HuMSC-Exos (25 μg/mL) were administered to the cell culture medium 24 h prior to irradiation and incubated at 37°C in a humidified atmosphere with 5% CO_2_ for subsequent experiments [24].

### 2.5. Quantitative Real-Time PCR (RT-qPCR) Analysis

Total RNA was isolated using TRIzol reagent (Invitrogen, USA) according to the manufacturer's instructions, and RNA purity was determined by measuring the A260/A280 ratio. Complementary DNA was then synthesized using the PrimeScript RT Reagent Kit (Takara, Japan). RT-qPCR was performed with SYBR Green PCR Master Mix (Vazyme, Nanjing, China) on an ABI 7500 StepOne Plus Real-Time PCR System (Applied Biosystems, USA), with β-actin serving as the internal reference gene. Primer sequences are listed in Table S1.

### 2.6. Western Blot

Western blotting was performed as previously described [25]. Briefly, cell lysates were prepared using radioimmunoprecipitation assay buffer (Yeasen, China) supplemented with protease and phosphatase inhibitors. Protein concentrations were determined with a BCA protein assay kit (Yeasen, China) and adjusted to 1 mg/mL. Equal amounts of protein (10 μg per lane) were denatured in Laemmli buffer (Yeasen, China) at 95 °C for 10 min, separated on 4%–20% gradient SDS–PAGE gels, and transferred onto polyvinylidene difluoride membranes (Merck KGaA, Darmstadt, Germany). Membranes were blocked with 5% nonfat milk and incubated overnight at 4 °C with primary antibodies against CD9 (13174S; 1:1000; Cell Signaling Technology), CD63 (ab134045; 1:1000; Abcam), TSG101 (ab133586; 1:1000; Abcam), Alix (ab275377; 1:1000; Abcam), β-actin (WL01372; 1:1000; Wanleibio, China), iNOS (WL0992a; 1:1000; Wanleibio, China), COX-2 (WL01750; 1:1000; Wanleibio, China), p-NF-κB (WL02169; 1:1000; Wanleibio, China), NF-κB (WL01980; 1:1000; Wanleibio, China), p-IκBα (WL02495; 1:1000; Wanleibio, China), IκBα (WL01936; 1:1000; Wanleibio, China), Keap1 (WL03285; 1:1000; Wanleibio, China), Nrf2 (WL02135; 1:1000; Wanleibio, China), Phospho-Nrf2 (ab76026; 1:1000; Abcam), Lamin B1 (WL01775; 1:1000; Wanleibio, China), and HO-1 (WL02400; 1:1000; Wanleibio, China). After washing, membranes were incubated with horseradish peroxidase (HRP)-conjugated secondary antibodies (WLA023; 1:3000; Wanleibio, China) for 1 h at room temperature. Protein bands were detected using the Pierce ECL Western Blotting Substrate Kit (Thermo Fisher Scientific, USA) and visualized on a ChemiScope imaging system (ChemiScope 5300, CLINX, China). Band intensities were quantified with GraphPad Prism software. For Nrf2 nuclear translocation analysis, nuclear and cytoplasmic fractions were isolated from HaCaTs using the NE-PER Nuclear and Cytoplasmic Extraction Kit (Thermo Fisher Scientific, USA) before Western blotting.

### 2.7. ELISA

HaCaTs were pretreated with HuMSC-Exos (25 μg/mL) for 24 h and subsequently exposed to UVB irradiation at a dose of 100 mJ/cm^2^. After an additional 24 h of incubation, the cell culture supernatants were harvested for cytokine analysis. The concentrations of IL-1β, TNF-α, and IL-6 were measured using commercial ELISA kits (R&D Systems, USA) according to the manufacturer's instructions. Absorbance was recorded at 450 nm using a microplate reader (Thermo Fisher Scientific, USA), and cytokine levels were calculated based on standard curves.

### 2.8. Cell Migration Assays

Scratch and transwell assays were performed to assess the migratory capacity of HaCaTs [26]. For the scratch assay, HaCaTs were seeded in 6-well plates at a density of 3.0 × 10^5^ cells/well and cultured until reaching confluence. Cells were pretreated with HuMSC-Exos (25 μg/mL) for 24 h, followed by UVB irradiation at 100 mJ/cm^2^. A linear wound was created across the cell monolayer using a sterile 200 μL pipette tip, after which the cells were cultured in serum-free medium for an additional 24 h. Images of the wound area were captured at 0 h and 24 h, and the migration distance was quantified using ImageJ software (National Institutes of Health, USA). For the transwell assay, HaCaTs subjected to the same treatments were seeded into the upper chamber of 24-well transwell inserts (pore size: 8 μm; Corning, USA) containing serum-free DMEM, while the lower chamber was filled with DMEM supplemented with 10% FBS to serve as a chemoattractant. After 24 h of incubation, cells remaining on the upper surface of the membrane were removed, whereas migrated cells on the lower surface were fixed with 4% paraformaldehyde, stained with 0.1% crystal violet, and visualized using an FSX100 microscope (Olympus, Japan). Migrated cells were quantified from randomly selected fields using ImageJ software.

### 2.9. Determination of ROS Levels in HaCaTs

HaCaTs were seeded into 12-well plates and subjected to the indicated treatments as described above. Intracellular ROS levels were measured using the fluorescent probe 2′,7′-dichlorofluorescein diacetate (DCFH-DA; 10 μM; Yeasen, 50101ES01, Shanghai, China). Briefly, cells were incubated with DCFH-DA at 37°C in the dark for 30 min, followed by one to two washes with serum-free medium to remove excess dye. Cell nuclei were counterstained with Hoechst 33342 (Beyotime, China), and fluorescence images were captured immediately using a fluorescence microscope (Olympus, Japan). The relative ROS levels were quantified by calculating the proportion of DCFH-DA–positive cells using ImageJ software (National Institutes of Health, USA).

### 2.10. Cell Immunofluorescence Staining

Cell immunofluorescence staining was performed as previously described [27]. Briefly, cells grown on coverslips were fixed with 4% paraformaldehyde for 15 min, rinsed three times with PBS, and blocked with 5% goat serum for 1 h at room temperature. Cells were then incubated overnight at 4 °C with an anti-Nrf2 primary antibody (GB113808-100; Servicebio, Wuhan, China), followed by incubation with an appropriate fluorophore-conjugated secondary antibody (Servicebio, Wuhan, China) for 50 min at room temperature in the dark. Nuclei were counterstained with DAPI (G1012; Servicebio, Wuhan, China) for 10 min. Fluorescence images were captured using a Zeiss LSM900 laser scanning confocal microscope (Carl Zeiss, Germany).

### 2.11. Transient Transfection

To inhibit Nrf2 activity, HaCaTs were transfected with Nrf2-specific siRNA (Shanghai GenePharma, China) using Lipofectamine 2000 (Thermo Fisher Scientific, USA), with non-targeting siRNA (NC-siRNA) serving as a negative control (NC). For miRNA modulation, transient overexpression or inhibition of miR-200a-3p was performed by transfecting miRNA mimics or inhibitors (GenePharma, China) using the same transfection reagent. Briefly, HaCaTs were seeded in 6-well plates at ~60% confluence and transfected with Nrf2 siRNA, NC-siRNA, miR-200a-3p mimics, or miR-200a-3p inhibitors for 24–48 h in serum-free medium. After transfection, cells were incubated with HuMSC-Exos (25 μg/mL) for 24 h and then exposed to UVB radiation. Transfection efficiency was assessed 48 h post-transfection by RT-qPCR and Western blot analysis [17].

### 2.12. RNA Sequencing Analysis

miRNA sequencing was performed by Aksomics (Shanghai, China). Total RNA was extracted from samples, and RNA quality and quantity were assessed using agarose gel electrophoresis and a Nanodrop spectrophotometer (Thermo Fisher Scientific, USA). Sequencing libraries were subsequently constructed and their quality evaluated using an Agilent 2100 Bioanalyzer (Agilent Technologies, USA). Libraries were denatured with 0.1 M NaOH to generate single-stranded DNA, followed by cyclic sequencing on an Illumina NextSeq 500 platform (Illumina, USA) according to the manufacturer's protocols. Target genes of highly expressed miRNAs were predicted using the miRDB database.

### 2.13. Dual-Luciferase Reporter Assay

Luciferase reporter vectors containing either the wild-type or mutant 3′ untranslated region (3′UTR) of Keap1, together with miR-200a-3p mimics, miR-423-5p mimics, or NC mimics, were obtained from GenePharma (China). HaCaTs were co-transfected with these vectors using Lipofectamine 2000 (Thermo Fisher Scientific, USA) according to the manufacturer's instructions. Forty-eight hours post-transfection, luciferase activity was measured using a Dual-Luciferase Reporter Assay System (Biotek Synergy, USA), and relative luciferase activity was calculated by normalizing firefly luciferase activity to Renilla luciferase activity.

### 2.14. Animals

All animal experiments were performed in accordance with the guidelines approved by the Medical Ethics Committee of Shanghai Changzheng Hospital. Healthy male C57BL/6 mice (6–8 weeks old, SPF grade) were purchased from Bikai Biological Technology Co., Ltd. (Shanghai, China) and maintained under standard laboratory conditions.

### 2.15. Animal Models and HuMSC-Exos Administration

UVB irradiation for the animal photodamage model was performed using PL-L36W/01/4P lamps (Philips, Poland). Male C57BL/6 mice were acclimated to the laboratory environment for 1 week prior to experimentation. Before UVB exposure, mice were anesthetized with 2% sodium pentobarbital, and the dorsal skin was depilated using depilatory cream (Nair, Church & Dwight) to remove hair. During irradiation, mice were housed individually to ensure uniform UVB exposure. The mice were randomly assigned to three groups (*n* = 5 per group): control, UVB + PBS, and UVB + Exosomes [28]. The control group received no treatment, whereas the UVB + PBS and UVB + Exosomes groups were exposed to UVB radiation daily for several minutes over five consecutive days, achieving a cumulative dose of 5 kJ/m^2^ [29]. In the UVB + Exosomes group, exosomes were administered immediately following each UVB session. Specifically, the dorsal skin was divided into four areas, with each area receiving a 25 μL subcutaneous injection of exosomes at 0.02 μg/μL, corresponding to a daily dose of 2 μg per mouse and a total of 10 μg over 5 days [24]. The UVB + PBS group received equal volumes of PBS following the same injection protocol. Twenty-four hours after the final treatment, skin condition was assessed, and tissue samples were collected for subsequent sectioning and further analyses.

### 2.16. Tissue Staining and Imaging

Hematoxylin and eosin (H&E) staining and Masson's trichrome staining were performed according to established protocols [20]. H&E staining was conducted using the H&E Staining Kit (Abcam, UK), while Masson's trichrome staining employed the Trichrome Stain (Masson) Kit (Sigma–Aldrich, USA). Immunohistochemistry was carried out as previously described [30]. Primary antibodies included anti-Nrf2 (WL02135; Wanleibio, China), anti-IL-1β (ab283818; Abcam, UK), anti-TNF-α (60291-1-Ig; Proteintech, China), and anti-IL-6 (26404-1-AP; Proteintech, China), with species-appropriate secondary antibodies (Abcam, UK). Stained tissue sections were scanned using a K-Viewer Digital Imaging System (KFBIO Tech, China), and quantification of marker expression was performed using ImageJ (National Institutes of Health, USA) and Image Pro Plus (IPP) software.

### 2.17. Statistical Analysis

Statistical analyses were performed using GraphPad Prism 9.0 (GraphPad Software, USA). All experiments were independently repeated at least three times, and data are presented as mean ± standard deviation (SD). Comparisons between two groups were conducted using Student's *t*-test, while comparisons among more than two groups were analyzed by one-way analysis of variance (ANOVA) followed by Bonferroni's post hoc test. Differences were considered statistically significant at *p* < 0.05.

## 3. Results

### 3.1. Characterization of HuMSCs and Exosomes

**Figure 1:**
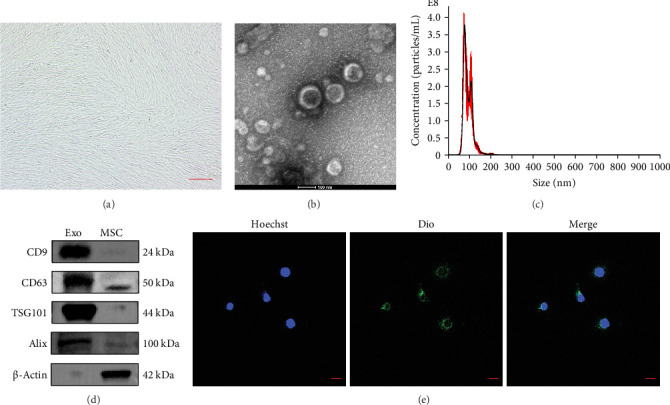
Characterization of HuMSCs and exosomes. (A) Morphology of HuMSCs observed under an inverted microscope. Scale bar: 100 μm. (B) TEM image of HuMSC-Exos showing characteristic cup-shaped morphology. Scale bar: 100 nm. (C) Particle size distribution of HuMSC-Exos analyzed by NTA. (D) Western blot analysis showing the expression of exosomal markers CD9, CD63, TSG101, and Alix in HuMSC-Exos. (E) Cellular uptake of DiO-labeled HuMSC-Exos (green) by HaCaTs; nuclei were counterstained with Hoechst (blue). Scale bar: 50 μm.

Under an inverted microscope, HuMSCs exhibited a typical spindle-shaped morphology (Figure 1A). HuMSC-Exos were isolated from early-passage cells (passages 2–6) using differential centrifugation. TEM revealed that the vesicles exhibited the characteristic cup-shaped morphology of exosomes, enclosed by a bilayer membrane (Figure 1B). NTA showed particle sizes ranging from 40 to 204 nm, with an average diameter of ~100 nm (Figure 1C). Western blotting confirmed the presence of canonical exosomal markers, including CD9, CD63, TSG101, and Alix, validating successful exosome isolation (Figure 1D). Furthermore, DiO-labeled HuMSC-Exos were efficiently internalized by HaCaTs, as observed by confocal microscopy (Figure 1E).

### 3.2. HuMSC-Exos Reduced UVB-Induced Inflammatory Cytokine Expression and Restored the Migratory Capacity of HaCaTs

**Figure 2:**
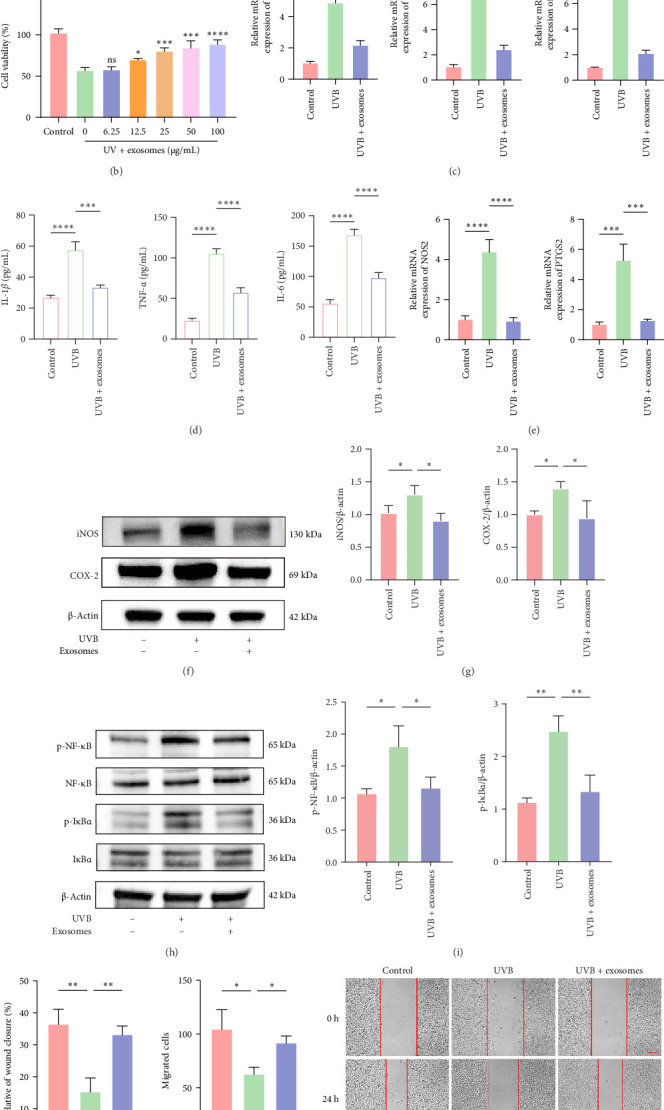
HuMSC-Exos attenuated inflammation and promoted cell migration in UVB-exposed HaCaTs. (A) RT-qPCR analysis of *IL1B*, *TNF*, and *IL6* mRNA levels in HaCaTs exposed to increasing doses of UVB (mJ/cm^2^). (B) CCK-8 assay evaluating the protective effects of HuMSC-Exos on HaCaTs viability. (C, D). RT-qPCR and ELISA analyses of inflammatory cytokine expression in HaCaTs under different treatments. (E–G) mRNA and protein expression levels of iNOS and COX-2 in HaCaTs under different treatments, along with corresponding grayscale analyses. (H, I) Western blot analysis and quantification of p-NF-κB, NF-κB, p-IκBα, and IκBα protein expression in HaCaTs following different treatments. (J, K) Scratch wound and transwell assays assessing the migratory capacity of HaCaTs after different treatments. Scale bar: 150 μm. Data are presented as mean ± SD (*n* = 3). (A, B) Treated groups vs. 0 group. ns: nonsignificant, *⁣*^*∗*^: *p* < 0.05, *⁣*^*∗∗*^: *p* < 0.01, *⁣*^*∗∗∗*^: *p* < 0.001, *⁣*^*∗∗∗∗*^: *p* < 0.0001).

To investigate the effects of UVB radiation on inflammatory responses, HaCaTs were exposed to increasing doses of UVB to establish an in vitro model of UV-induced damage. RT-qPCR analysis revealed a dose-dependent upregulation of inflammatory cytokines, including *IL1B*, *TNF*, and *IL6*, with high expression observed at 100 mJ/cm^2^ (Figure 2A), which was therefore selected for subsequent experiments. Pretreatment with HuMSC-Exos prior to UVB exposure significantly restored cell viability, as determined by CCK-8 assays, with 25 μg/mL showing a statistically significant effect (Figure 2B). RT-qPCR and ELISA analyses demonstrated that HuMSC-Exos markedly attenuated UVB-induced upregulation of IL-1β, TNF-α, and IL-6 at both the mRNA and protein levels (Figure 2C,D). Furthermore, HuMSC-Exos reduced the expression of inflammatory enzymes iNOS (*NOS2*) and COX-2 (*PTGS2*) (Figure 2E–G) and suppressed NF-κB pathway activation, as evidenced by decreased phosphorylation of NF-κB and IκBα (Figure 2H,I). Functional assays revealed that UVB exposure impaired HaCaTs migration, which was effectively restored by HuMSC-Exos in both scratch and transwell assays (Figure 2J,K). Collectively, these results indicate that HuMSC-Exos mitigate UVB-induced inflammation and restore the migratory capacity of keratinocytes.

### 3.3. HuMSC-Exos Attenuated UVB-Induced Oxidative Stress in HaCaTs by Activating the Nrf2 Signaling Pathway

**Figure 3:**
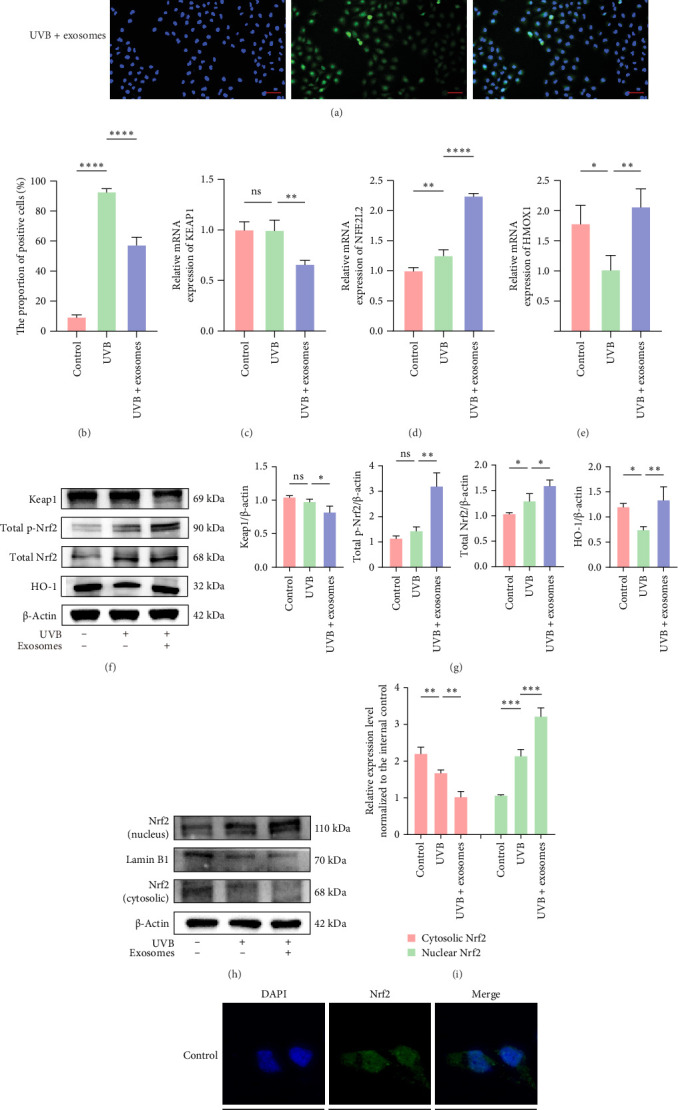
HuMSC-Exos attenuated oxidative stress in UVB-exposed HaCaTs by activating the Nrf2 signaling pathway. (A, B) DCFH-DA staining and quantification of ROS levels in HaCaTs after different treatments (blue: Hoechst; green: ROS; scale bar: 100 μm). (C–G) RT-qPCR and Western blot analyses of Keap1, Nrf2 (p-Nrf2 and total Nrf2), and HO-1 expression, with corresponding grayscale quantification. (H, I). Western blot and quantification of nuclear and cytoplasmic Nrf2 protein levels. (J) Immunofluorescence staining showing Nrf2 localization (blue: DAPI; green: Nrf2). Scale bar: 50 μm. Data are presented as mean ± SD (*n* = 3). (ns: nonsignificant, *⁣*^*∗*^: *p* < 0.05, *⁣*^*∗∗*^: *p* < 0.01, *⁣*^*∗∗∗*^: *p* < 0.001, *⁣*^*∗∗∗∗*^: *p* < 0.0001).

ROS, partially reduced oxygen molecules generated during the electron transport chain, acts as key regulators of acute inflammatory responses. UVB radiation has been reported to induce ROS production, thereby activating multiple intracellular signaling pathways [31, 32]. Using DCFH-DA fluorescence analysis, we confirmed that pretreatment with HuMSC-Exos significantly attenuated UVB-induced ROS accumulation in HaCaTs (Figure 3A,B). Considering the pivotal role of the redox-sensitive transcription factor Nrf2 in regulating cellular antioxidant defenses through upregulation of a network of antioxidant genes and mitigating photoaging [33], we hypothesized that the antioxidant effects of HuMSC-Exos involve Nrf2 activation. To test this, we evaluated Nrf2 mRNA levels, total and phosphorylated protein expression, and nuclear translocation. HuMSC-Exos markedly increased both the transcriptional and protein levels of Nrf2 and its downstream target HO-1, enhanced Nrf2 phosphorylation, and concurrently decreased Keap1 mRNA and protein levels compared with the UVB group (Figure 3C–I). Immunofluorescence analysis further demonstrated that HuMSC-Exos promoted Nrf2 nuclear translocation (Figure 3J). Additionally, RT-qPCR analysis of Nrf2 downstream targets, including catalase (CAT), NQO1, and superoxide dismutase 2 (SOD2), revealed significantly elevated mRNA expression in exosome-treated cells relative to the UVB group (Figure S1). Together, these results indicate that HuMSC-Exos exert antioxidant effects by activating the Nrf2 signaling pathway, thereby enhancing cellular defense mechanisms against UVB-induced oxidative stress.

### 3.4. Nrf2 Knockdown Attenuated the Anti-Inflammatory Effects of HuMSC-Exos

**Figure 4:**
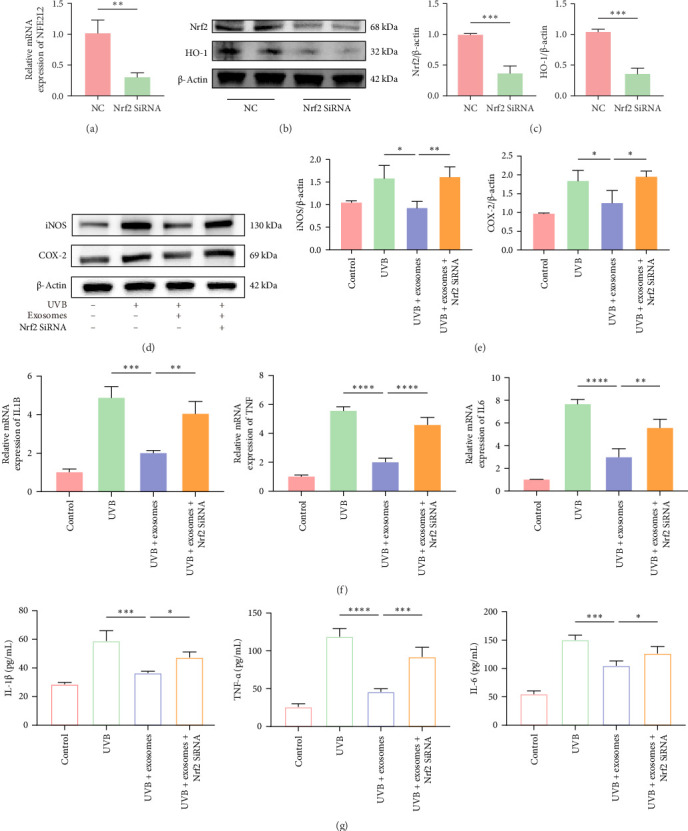
Knockdown of Nrf2 attenuated the anti-inflammatory effects of HuMSC-Exos. (A) RT-qPCR analysis confirming the knockdown efficiency of Nrf2. (B, C) Western blot and grayscale analysis showing Nrf2 and HO-1 protein expression. (D, E) Protein levels of iNOS and COX-2 in HaCaTs following different treatments and corresponding grayscale quantification. (F) RT-qPCR analysis of *IL1B*, *TNF*, and *IL6* mRNA expression in HaCaTs under various treatments. (G) ELISA quantification of IL-1β, TNF-α, and IL-6 levels in cell culture supernatants. Data are presented as mean ± SD (*n* = 3). (ns: nonsignificant, *⁣*^*∗*^*p* < 0.05, *⁣*^*∗∗*^*p* < 0.01, *⁣*^*∗∗∗*^*p* < 0.001, *⁣*^*∗∗∗∗*^*p* < 0.0001).

To examine the role of Nrf2 in mediating the anti-inflammatory effects of HuMSC-Exos in HaCaTs, Nrf2 expression was silenced using siRNA. Efficient knockdown was confirmed by RT-qPCR and Western blot, showing marked reductions in both Nrf2 mRNA and protein levels compared with the NC group. Consequently, expression of the downstream target HO-1 was significantly decreased (Figure 4A–C). Nrf2 silencing also resulted in elevated protein levels of iNOS and COX-2 (Figure 4D,E), accompanied by upregulation of proinflammatory cytokine mRNAs, including *IL1B*, *TNF*, and *IL6* (Figure 4F), which was further confirmed by ELISA analysis of IL-1β, TNF-α, and IL-6 in culture supernatants (Figure 4G). These findings indicate that Nrf2 is critical for the anti-inflammatory activity of HuMSC-Exos, and its knockdown significantly attenuates the protective effects of exosomes against UVB-induced inflammation in HaCaTs.

### 3.5. HuMSC-Exos Modulate the Nrf2 Signaling Pathway in HaCaTs via miR-200a-3p-Mediated Targeting of Keap1

**Figure 5:**
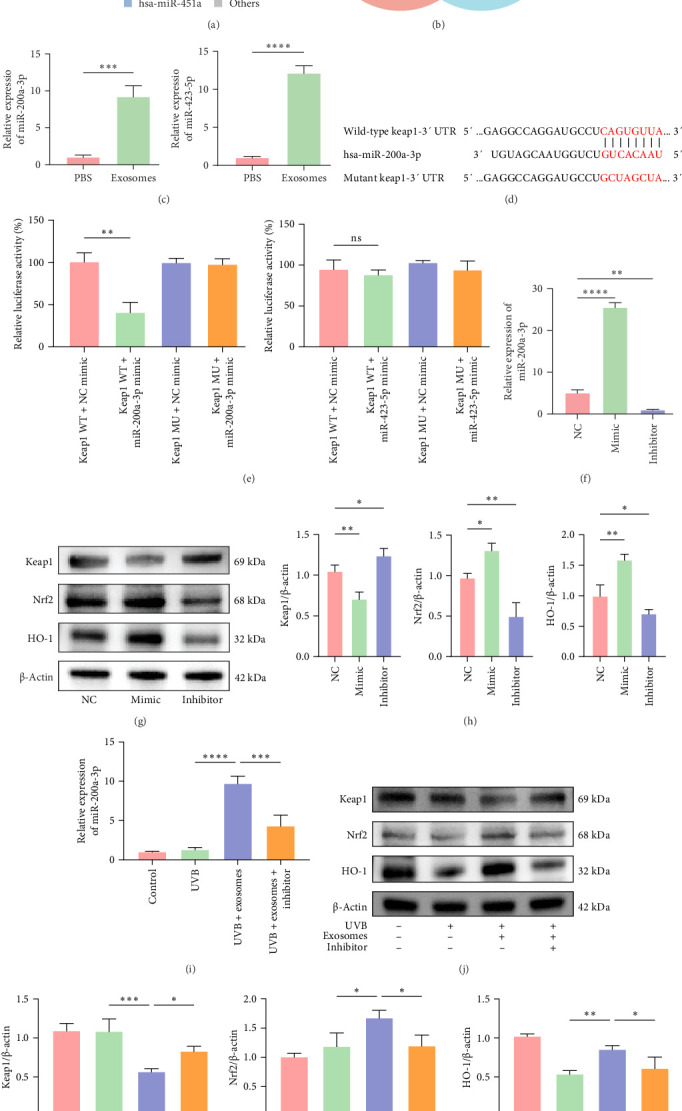
HuMSC-Exos regulated the Nrf2 signaling pathway by delivering miR-200a-3 p targeting Keap1. (A, B) miRNA sequencing analysis showing relative miRNA proportions and Venn diagram illustrating intersecting miRNAs. (C) RT-qPCR validation of miR-200a-3 p and miR-423-5 p expression. (D, E) Dual-luciferase reporter assays confirming the direct binding of miR-200a-3 p to Keap1 3′UTR. (F–H) RT-qPCR and Western blot analysis of Keap1, Nrf2, and HO-1 expression in HaCaTs transfected with NC, miR-200a-3 p mimics, or inhibitors, and grayscale quantification of protein bands. (I–K) Validation of miR-200a-3 p effects on Keap1/Nrf2/HO-1 expression in differently treated HaCaTs by RT-qPCR, Western blot, and grayscale analysis. Data are presented as mean ± SD (*n* = 3). (ns: nonsignificant, *⁣*^*∗*^*p* < 0.05, *⁣*^*∗∗*^*p* < 0.01, *⁣*^*∗∗∗*^*p* < 0.001, *⁣*^*∗∗∗∗*^*p* < 0.0001).

MiRNAs are short noncoding RNAs that regulate gene expression by binding to target mRNAs and can be transferred between cells via exosomes [34]. In this study, HuMSC-Exos suppressed Keap1 expression and promoted Nrf2 nuclear translocation, suggesting that specific exosomal miRNAs may target Keap1 mRNA, leading to its degradation and subsequent activation of Nrf2 signaling. To identify candidate miRNAs, RNA sequencing was performed, revealing 613 distinct miRNAs in HuMSC-Exos. The top five most abundant miRNAs are shown in Figure 5A. Target prediction using the miRDB database identified 30 miRNAs potentially targeting Keap1 (NCBI Gene ID: 9817) (Figure S2), seven of which overlapped with our sequencing results: hsa-miR-3184-5p, hsa-miR-4449, hsa-miR-1269a, hsa-miR-141-3p, hsa-miR-200a-3p, hsa-miR-423-5p, and hsa-let-7c-3p (Figure 5B). Among these, miR-200a-3p and miR-423-5p exhibited the highest expression levels and were selected for further functional validation (Figure S3). Compared with PBS-treated HaCaTs, HuMSC-Exos significantly increased miR-200a-3p and miR-423-5p levels (Figure 5C). Dual-luciferase reporter assays demonstrated that miR-200a-3p directly binds the 3′UTR of Keap1 mRNA, as indicated by a significant reduction in luciferase activity of the wild-type 3′UTR, whereas mutation of the binding site abolished this effect (Figure 5D,E). In contrast, miR-423-5p did not affect luciferase activity of either the wild-type or mutant 3′UTR, indicating that it does not target Keap1. Functional experiments using miR-200a-3p mimics and inhibitors further confirmed these results: mimics elevated miR-200a-3p expression, reduced Keap1 protein levels, and increased Nrf2 and HO-1 expression, whereas inhibitors produced the opposite effects (Figure 5F–H). Notably, co-treatment of HuMSC-Exos with miR-200a-3p inhibitors in UVB-irradiated HaCaTs attenuated the exosome-mediated modulation of Keap1, Nrf2, and HO-1 (Figure 5I–K). Collectively, these findings indicate that HuMSC-Exos regulate the Nrf2 signaling pathway through miR-200a-3p-mediated targeting of Keap1, revealing a key molecular mechanism underlying their antioxidant and cytoprotective effects in UVB-induced skin injury.

### 3.6. MiR-200a-3p Mediates Anti-Inflammatory and Antioxidant Effects via the Nrf2 Signaling Pathway

**Figure 6:**
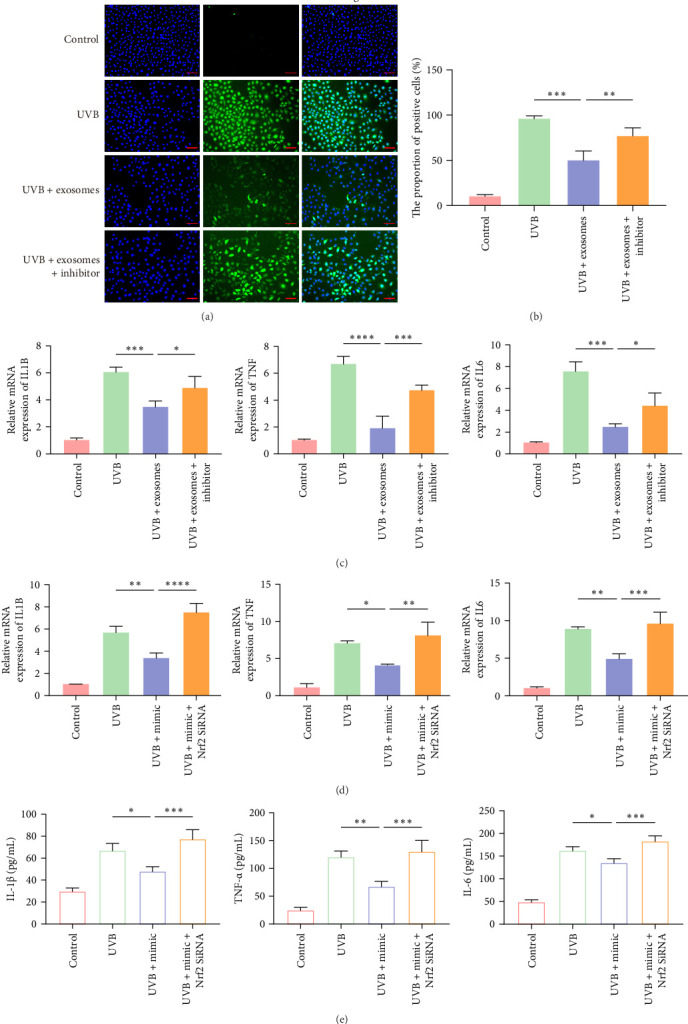
miR-200a-3 p exerted anti-inflammatory and antioxidant effects via the Nrf2 signaling pathway. (A, B) DCFH-DA staining and quantification of ROS levels in HaCaTs after different treatments (blue: Hoechst, green: ROS; scale bar: 100 μm). (C–E) RT-qPCR and ELISA analysis of IL-1β, TNF-α, and IL-6 mRNA and protein levels in HaCaTs under various treatments. Data are presented as mean ± SD (*n* = 3). (ns: nonsignificant, *⁣*^*∗*^*p* < 0.05, *⁣*^*∗∗*^*p* < 0.01, *⁣*^*∗∗∗*^*p* < 0.001, *⁣*^*∗∗∗∗*^*p* < 0.0001).

To investigate whether miR-200a-3p mediates the antioxidative and anti-inflammatory effects of HuMSC-Exos through the Nrf2 signaling pathway, UVB-irradiated HaCaTs were treated with HuMSC-Exos in the presence or absence of miR-200a-3p inhibitors. DCFH-DA fluorescence analysis revealed that inhibition of miR-200a-3p significantly attenuated the ROS-scavenging effects of HuMSC-Exos (Figure 6A,B). Consistently, assessment of inflammatory cytokines showed that miR-200a-3p inhibitors partially reversed the anti-inflammatory effects of HuMSC-Exos, resulting in elevated levels of *IL1B*, *TNF*, and *IL6* compared with the exosome-treated group (Figure 6C). To further confirm the role of miR-200a-3p in modulating inflammation via Nrf2, HaCaTs were transfected with miR-200a-3p mimics, with or without Nrf2 knockdown. Transfection with miR-200a-3p mimics markedly reduced IL-1β, TNF-α, and IL-6 expression in UVB-exposed HaCaTs. However, in Nrf2-silenced cells, miR-200a-3p mimics failed to suppress these inflammatory cytokines, and inflammatory responses were even exacerbated compared with the UVB group (Figure 6D,E). Collectively, these results indicate that miR-200a-3p mediates the antioxidative and anti-inflammatory effects of HuMSC-Exos predominantly through activation of the Nrf2 signaling pathway, highlighting its critical role in exosome-mediated cytoprotection against UVB-induced oxidative stress and inflammation.

### 3.7. HuMSC-Exos Attenuated Inflammatory Responses, Reduced Tissue Damage, and Modulated the Nrf2 Signaling Pathway in UVB-Irradiated Mouse Skin

**Figure 7:**
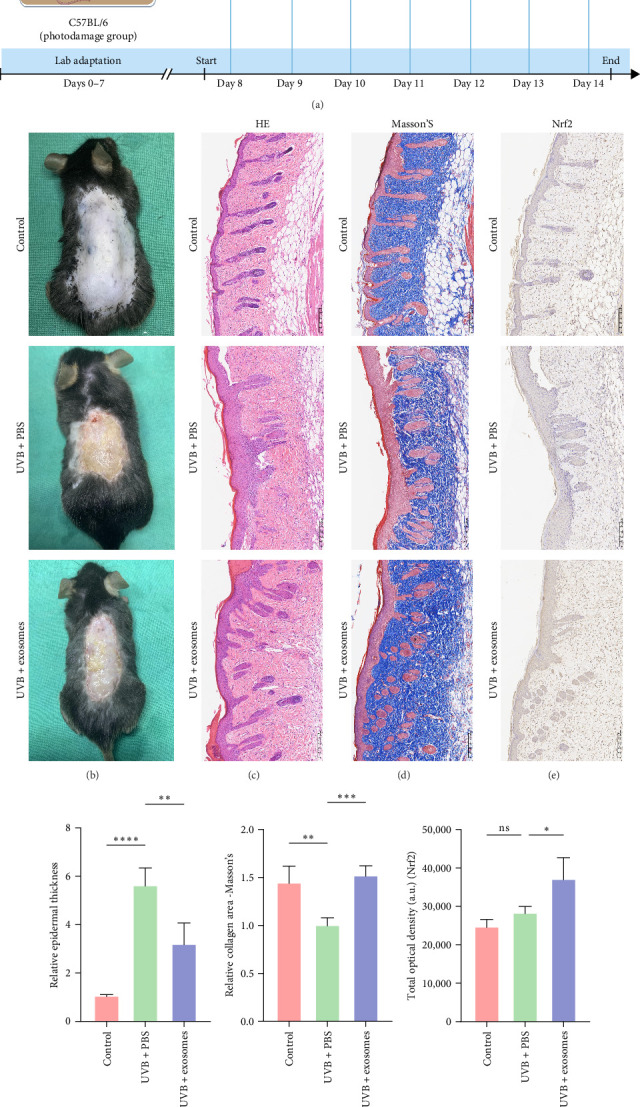
Intradermal injection of HuMSC-Exos alleviated UVB-induced skin inflammation, reduced tissue damage, and enhanced Nrf2 expression in vivo. (A) Schematic diagram of the animal experimental design. (B) Representative images showing dorsal skin morphology in different treatment groups (*n* = 5/group). (C–E) H&E, Masson's trichrome, and Nrf2 immunohistochemical staining of mouse skin tissues under various treatments (scale bar: 200 μm). (F–H) Quantification of epidermal thickness, collagen area, and Nrf2 immunostaining optical density (*n* = 4/group). (ns: nonsignificant, *⁣*^*∗*^*p* < 0.05, *⁣*^*∗∗*^*p* < 0.01, *⁣*^*∗∗∗*^*p* < 0.001, *⁣*^*∗∗∗∗*^*p* < 0.0001).

**Figure 8:**
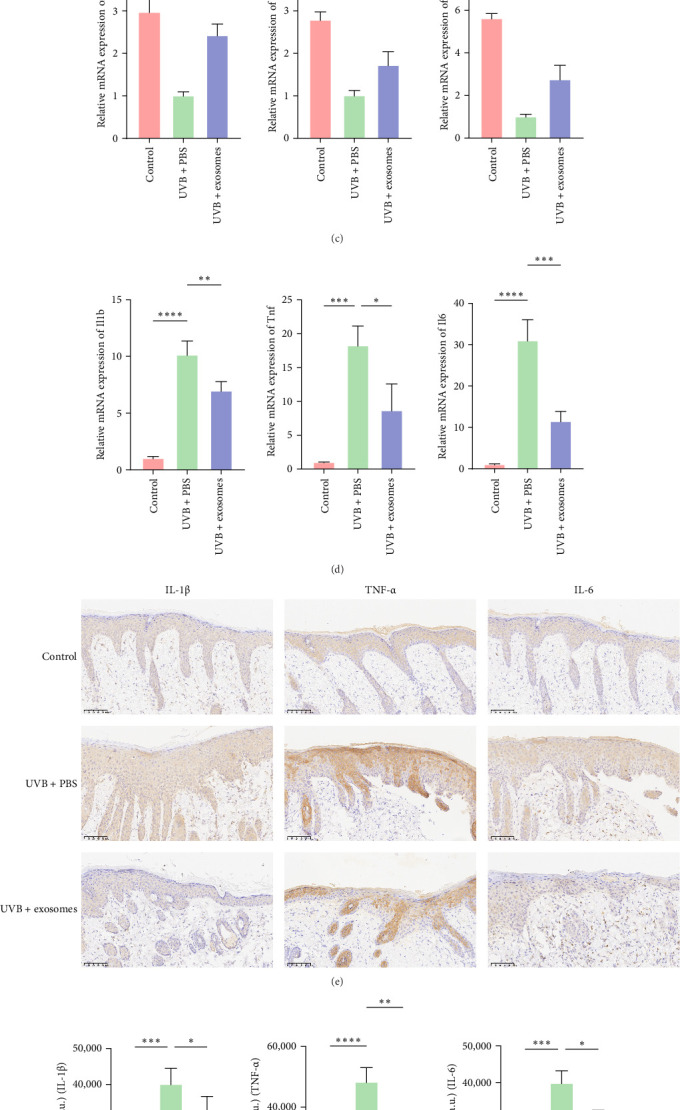
HuMSC-Exos activated the Nrf2 signaling pathway, upregulated antioxidant gene expression, and attenuated inflammation in UVB-irradiated mouse skin. (A, B) Western blot analysis and grayscale quantification of Keap1, Nrf2, and HO-1 expression in mouse skin after different treatments (*n* = 3/group). (C) RT-qPCR detection of antioxidant genes *Cat*, *Nqo1*, and *Sod2* in skin tissues (*n* = 3/group). (D–F) RT-qPCR and immunohistochemical analysis of IL-1β, TNF-α, and IL-6 expression, with quantitative optical density measurements (*n* = 4/group; scale bar: 100 μm). (ns: nonsignificant, *⁣*^*∗*^*p* < 0.05, *⁣*^*∗∗*^*p* < 0.01, *⁣*^*∗∗∗*^*p* < 0.001, *⁣*^*∗∗∗∗*^*p* < 0.0001).

To evaluate the protective and anti-inflammatory effects of HuMSC-Exos against UVB-induced skin injury, a murine UVB photodamage model was established (Figure 7A). Mice receiving intradermal injections of HuMSC-Exos displayed markedly improved dorsal skin appearance compared with UVB-exposed mice treated with PBS (Figure 7B). Histological analysis revealed a significant reduction in epidermal thickness in exosome-treated mice relative to the UVB + PBS group (Figure 7C,F). Masson's trichrome staining further indicated that collagen content and structural integrity were largely preserved following exosome administration (Figure 7D,G). Immunohistochemical staining demonstrated a substantial increase in Nrf2-positive cells and total optical density in the exosome-treated group, suggesting activation of the Nrf2 signaling pathway (Figure 7E,H). Concordantly, Western blot analysis showed elevated Nrf2 and HO-1 protein levels and decreased Keap1 expression in the exosome-treated group compared with UVB + PBS mice (Figure 8A,B). RT-qPCR further confirmed upregulation of antioxidant genes, including *Cat*, *Nqo1*, and *Sod2* (Figure 8C), whereas mRNA levels of proinflammatory cytokines *Il1b*, *Tnf*, and *Il6* were significantly reduced (Figure 8D), consistent with immunohistochemical observations (Figure 8E,F). Collectively, these results indicate that HuMSC-Exos confer robust cytoprotective and anti-inflammatory effects in vivo, likely via activation of the Nrf2 pathway, enhancement of antioxidant defenses, and suppression of inflammatory cytokine expression.

## 4. Discussion

UVB radiation is a major environmental stressor that promotes ROS accumulation and activates inflammatory signaling pathways in keratinocytes, culminating in cellular dysfunction and skin injury. Excessive ROS not only damages cellular macromolecules such as lipids, proteins, and DNA but also serves as a signaling molecule that further amplifies inflammatory responses by activating transcription factors such as NF-κB and AP-1 [35]. This oxidative and inflammatory milieu disrupts the homeostasis of keratinocytes, leading to impaired proliferation, reduced migration capacity, and increased apoptosis, which collectively compromise the integrity of the epidermal barrier. A recent study has shown that UVB irradiation reduces HaCaTs cell viability by inducing excessive ROS production and lipid peroxidation, triggering oxidative stress, inflammation (upregulation of TNF-α, COX-2, iNOS, IL-1β), and apoptosis (altered Bcl-2, Bax, and Caspase-3 expression), ultimately impairing keratinocyte homeostasis [6]. In line with these findings, our study shows that UVB exposure markedly increased ROS levels and upregulated proinflammatory mediators, including the cytokines IL-1β, TNF-α, and IL-6, as well as the inflammation-associated enzymes iNOS and COX-2, while activating the NF-κB signaling pathway. These changes were accompanied by impaired migratory capacity in HaCaTs, a critical process for skin repair.

In recent years, accumulating evidence has highlighted the potent protective effects of exosomes against UV-induced skin injury. For instance, Luo et al. [36] reported that milk-derived exosomes loaded SS31 alleviated UV-induced photodamage by modulating SOD and glutathione levels and suppressing the expression of pMAPK, AP-1, MMP-1, and MMP-3. Similarly, Liu et al. [28] demonstrated that exosomes derived from hypoxic pretreated adipose-derived stem cells mitigated UV-induced oxidative stress, inflammatory cytokine expression, and collagen degradation by delivering GLRX5 to inhibit ferroptosis. Moreover, Sun et al. [37] revealed that aloe-derived exosome-like nanoparticles protected against oxidative stress and DNA damage by activating the Nrf2/ARE signaling pathway, thereby delaying photoaging. Consistent with these findings, our study demonstrated that UVB irradiation triggers a compensatory upregulation of Nrf2 expression in keratinocytes as part of the intrinsic defense mechanism against oxidative stress. However, excessive and sustained UVB exposure leads to persistent oxidative stress, resulting in the depletion of key antioxidant enzymes, including HO-1, CAT, NQO1, and SOD2, ultimately exacerbating oxidative injury and inflammation. Remarkably, HuMSC-Exos pretreatment promoted Nrf2 phosphorylation and nuclear translocation, thereby activating the Nrf2 signaling pathway and restoring the expression of antioxidant enzymes. Nrf2 silencing with siRNA attenuated the anti-inflammatory effects of HuMSC-Exos, underscoring the pivotal role of Nrf2 in mediating their protective functions. Furthermore, in UVB-irradiated C57BL/6 mouse models, HuMSC-Exos treatment enhanced the expression of Nrf2, HO-1, CAT, NQO1, and SOD2 while suppressing Keap1 and reducing proinflammatory cytokine levels. HuMSC-Exos also attenuated UVB-induced epidermal thickening and collagen degradation. Notably, clinical studies have also begun to explore the application of exosomes in human skin, further advancing their translation from basic research to clinical practice. For example, Nguyen et al. [38] reported in a prospective clinical trial that topical application of adipose-derived stem cell exosomes for eight consecutive weeks significantly improved skin texture, enhanced skin elasticity and hydration, and reduced roughness and mean pore volume, demonstrating promising photoprotection and skin repair effects. This finding not only provides preliminary evidence for the clinical application of exosomes in the field of anti-photoaging but also suggests their broad potential in skin esthetics and the treatment of skin injuries.

Exosomes are enriched with diverse bioactive molecules, including miRNAs, proteins, and lipids, which may synergistically modulate cellular redox homeostasis and inflammatory responses. For instance, Yan et al. [39] demonstrated that bone marrow mesenchymal stem cell-derived exosomal miR-29b-3p attenuates photoaging by regulating MMP-2 expression and reducing oxidative stress. Likewise, Gao et al. [40] reported that exosomes overexpressing miR-1246 alleviate UVB-induced inflammatory responses via modulation of the IκBα/NF-κB signaling pathway. In our study, we identified that HuMSC-Exos deliver specific miRNAs, such as miR-200a-3p, which directly targets Keap1 to promote Nrf2 nuclear translocation and transcriptional activation of downstream antioxidant enzymes, thereby exerting dual regulatory effects on oxidative stress and inflammation at the molecular level. Interestingly, when Nrf2 expression was silenced using siRNA in UVB-irradiated HaCaTs, transfection with miR-200a-3p mimics resulted in higher expression levels of inflammatory cytokines compared with exosome pretreatment. This finding suggests that, in addition to miR-200a-3p, other functional components within HuMSC-Exos may contribute to their anti-inflammatory effects. Moreover, as miR-200a-3p has been implicated in diverse biological processes such as cell proliferation, migration, and epithelial–mesenchymal transition [41, 42], comprehensive studies integrating high-throughput sequencing, multi-omics analyses, and functional assays are warranted to elucidate both the individual and synergistic roles of exosomal cargo in regulating cellular physiology and pathology.

## 5. Conclusion

In this study, we demonstrated that UVB radiation induces oxidative stress, upregulates inflammatory mediators, activates the NF-κB signaling pathway, and impairs the migratory capacity of HaCaTs, whereas pretreatment with HuMSC-Exos effectively mitigates these detrimental effects. Mechanistically, HuMSC-Exos downregulate Keap1 expression, enhance Nrf2 and HO-1 expression, increase Nrf2 phosphorylation, and promote its nuclear translocation in UVB-exposed HaCaTs. Concurrently, HuMSC-Exos suppress NF-κB pathway activation by reducing the phosphorylation of NF-κB and IκBα. In addition, Nrf2 knockdown significantly attenuated the anti-inflammatory effects of HuMSC-Exos. Further analyses identified miR-200a-3p as a key effector within HuMSC-Exos, which directly targets Keap1 to facilitate Nrf2 activation and nuclear translocation, thereby upregulating cytoprotective genes such as HO-1 and collectively reducing oxidative stress and inflammatory responses. In UVB-induced skin injury models in mice, HuMSC-Exos alleviated inflammation, reduced tissue damage, and modulated the Nrf2 signaling cascade. Taken together, these findings reveal a novel protective mechanism whereby HuMSC-Exos deliver miR-200a-3p to attenuate UVB-induced oxidative stress and inflammation through the Keap1-Nrf2 pathway, ultimately improving tissue resilience against damage.

## Data Availability

The data that support the findings of this study are available from the corresponding author upon reasonable request.

## Nomenclature

UV: Ultraviolet
HuMSC-Exos: Human umbilical cord mesenchymal stem cell-derived exosomes
HaCaTs: Human immortalized keratinocytes
ROS: Reactive oxygen species
COX-2: Cyclooxygenase-2
iNOS: Inducible nitric oxide synthase
CAT: Catalase
NQO1: NAD(P)H quinone dehydrogenase 1
SOD2: Superoxide dismutase 2
Nrf2: Nuclear factor erythroid 2-related factor 2
Keap1: Kelch-like ECH-associated protein 1
AREs: Antioxidant response elements
HO-1: Heme oxygenase-1
miRNAs: MicroRNAs
CCK-8: Cell counting kit-8
FBS: Fetal bovine serum
DMEM: Dulbecco's modified eagle medium
TEM: Transmission electron microscopy
NTA: Nanoparticle tracking analysis
RT-qPCR: Quantitative real-time PCR
DCFH-DA: 2′,7′-dichlorofluorescein diacetate
H&E: Hematoxylin and eosin

## Supporting Information

Supporting Information Figure S1: CAT, NQO1, and SOD2 mRNA expression in HaCaTs after different treatments. Figure S2: Keap1 is predicted to be targeted by 30 miRNAs in miRDB. Figure S3: Expression levels of seven miRNAs in sequencing data. Table S1: RT-qPCR primer sequences.

## References

[1] Ghajar-Rahimi G., Yusuf N., Xu H. (2025). Ultraviolet Radiation-Induced Tolerogenic Dendritic Cells in Skin: Insights and Mechanisms. *Cells*.

[2] Gui Q., Ding N., Yao Z. (2024). Extracellular Vesicles Derived From Mesenchymal Stem Cells: The Wine in Hebe’s Hands to Treat Skin Aging. *Precision Clinical Medicine*.

[3] Yang S., Park S. H., Oh S. W. (2022). Antioxidant Activities and Mechanisms of Tomentosin in Human Keratinocytes. *Antioxidants*.

[4] Chen Q., Lin W., Tang Y. (2025). Curcumin Targets YAP1 to Enhance Mitochondrial Function and Autophagy, Protecting Against UVB-Induced Photodamage. *Frontiers in Immunology*.

[5] Wu P. Y., Lin T. Y., Hou C. W. (2019). 1,2-Bis[(3-Methoxyphenyl)Methyl]Ethane-1,2-Dicarboxylic Acid Reduces UVB-Induced Photodamage In Vitro and In Vivo. *Antioxidants*.

[6] Ding C., Peng X., Yang J. (2023). Rg3-Loaded P407/CS/HA Hydrogel Inhibits UVB-Induced Oxidative Stress, Inflammation and Apoptosis in HaCaT Cells. *Biomedicine & Pharmacotherapy*.

[7] St Denis A., Simonette R., Rady P. L., Tyring S. K. (2025). The Role of Prostaglandin Pathway and EP Receptors in Skin Cancer Development. *International Journal of Dermatology*.

[8] Camillo L., Zavattaro E., Veronese F., Gironi L. C., Cremona O., Savoia P. (2024). Ex Vivo Analysis of Cell Differentiation, Oxidative Stress, Inflammation, and DNA Damage on Cutaneous Field Cancerization. *International Journal of Molecular Sciences*.

[9] Liu J., Yang X., Huang X. (2025). Antioxidant and Anti-Inflammatory Effects of Crude *Gastrodia elata* Polysaccharides in UVB-Induced Acute Skin Damage. *Antioxidants*.

[10] Manavi M. A., Mohammad Jafari R., Shafaroodi H., Dehpour A. R. (2025). The Keap1/Nrf2/ARE/HO-1 Axis in Epilepsy: Crosstalk Between Oxidative Stress and Neuroinflammation. *International Immunopharmacology*.

[11] Kobayashi E. H., Suzuki T., Funayama R. (2016). Nrf2 Suppresses Macrophage Inflammatory Response by Blocking Proinflammatory Cytokine Transcription. *Nature Communications*.

[12] Li H., Xu N., Li S. (2025). Exosomes Derived From Umbilical Cord Mesenchymal Stem Cells Alleviate Jaw Bone Marrow Mesenchymal Stem Cells Senescence and Restore Osteogenic Differentiation Potential. *Stem Cell Research & Therapy*.

[13] Fang J., Nan L., Song K. (2024). Application and Progress of Bionic Scaffolds in Nerve Repair: A Narrative Review. *Advanced Technology in Neuroscience*.

[14] Yu A., Zhang Y., Zhong S., Yang Z., Xie M. (2025). Human Umbilical Cord Mesenchymal Stem Cell-Derived Exosomes Enhance Follicular Regeneration in Androgenetic Alopecia via Activation of Wnt/β-Catenin Pathway. *Stem Cell Research & Therapy*.

[15] Zhang H., Xiao X., Wang L. (2024). Human Adipose and Umbilical Cord Mesenchymal Stem Cell-Derived Extracellular Vesicles Mitigate Photoaging via TIMP1/Notch1. *Signal Transduction and Targeted Therapy*.

[16] Yan J., Gu Q., Li J. (2025). MS-275 Facilitates Osseointegration in Osteoporotic Rats by Mitigating Oxidative Stress via Activation of the miR-200a/Keap1/Nrf2 Signaling Pathway. *Redox Report*.

[17] Peng S., Shen L., Yu X. (2023). MiR-200a Attenuated Oxidative Stress, Inflammation, and Apoptosis in Dextran Sulfate Sodium-Induced Colitis Through Activation of Nrf2. *Frontiers in Immunology*.

[18] Hua T., Yang M., Song H. (2022). Huc-MSCs-Derived Exosomes Attenuate Inflammatory Pain by Regulating Microglia Pyroptosis and Autophagy via the miR-146a-5p/TRAF6 Axis. *Journal of Nanobiotechnology*.

[19] Xiong Y., Tang R., Xu J. (2022). Tongxinluo-Pretreated Mesenchymal Stem Cells Facilitate Cardiac Repair via Exosomal Transfer of miR-146a-5p Targeting IRAK1/NF-κB p65 Pathway. *Stem Cell Research & Therapy*.

[20] Ding N., Fu X., Gui Q. (2024). Biomimetic Structure Hydrogel Loaded With Long-Term Storage Platelet-Rich Plasma in Diabetic Wound Repair. *Advanced Healthcare Materials*.

[21] Yao X., Zhan L., Yan Z. (2023). Non-Electric Bioelectrical Analog Strategy by a Biophysical-Driven Nano-Micro Spatial Anisotropic Scaffold for Regulating Stem Cell Niche and Tissue Regeneration in a Neuronal Therapy. *Bioactive Materials*.

[22] Feng Z., Qin Y., Huo F. (2022). NMN Recruits GSH to Enhance GPX4-Mediated Ferroptosis Defense in UV Irradiation Induced Skin Injury. *Biochimica et Biophysica Acta (BBA) - Molecular Basis of Disease*.

[23] Liu T., Xia Q., Lv Y. (2023). ErZhiFormula Prevents UV-Induced Skin Photoaging by Nrf2/HO-1/NQO1 Signaling: An in Vitro and in Vivo Studies. *Journal of Ethnopharmacology*.

[24] Wang T., Jian Z., Baskys A. (2020). MSC-Derived Exosomes Protect Against Oxidative Stress-Induced Skin Injury via Adaptive Regulation of the NRF2 Defense System. *Biomaterials*.

[25] Gui Q., Luo W., Wu D. (2025). Construction and Validation of a Lipid Metabolism-Related Genes Prognostic Signature for Skin Cutaneous Melanoma. *Biochemical and Biophysical Research Communications*.

[26] Yan Z., Ye T., Yang L. (2023). Nanobiology Dependent Therapeutic Convergence Between Biocompatibility and Bioeffectiveness of Graphene Oxide Quantum Dot Scaffold for Immuno-Inductive Angiogenesis and Nerve Regeneration. *Advanced Functional Materials*.

[27] Ji R., Jia F., Chen X., Gao Y., Yang J. (2023). Carnosol Inhibits KGN Cells Oxidative Stress and Apoptosis and Attenuates Polycystic Ovary Syndrome Phenotypes in Mice Through Keap1-Mediated Nrf2/HO-1 Activation. *Phytotherapy Research*.

[28] Liu Y., Wang Y., Yang M. (2024). Exosomes From Hypoxic Pretreated ADSCs Attenuate Ultraviolet Light-Induced Skin Injury via GLRX5 Delivery and Ferroptosis Inhibition. *Photochemical & Photobiological Sciences*.

[29] Vats K., Kruglov O., Mizes A. (2021). Keratinocyte Death by Ferroptosis Initiates Skin Inflammation After UVB Exposure. *Redox Biology*.

[30] Wen J., Li L., Ou D. (2025). Higenamine Protects Against Doxorubicin-Induced Heart Failure by Attenuating Ferroptosis via Modulating the Nrf2/GPX4 Signaling Pathway. *Phytomedicine*.

[31] Sun J. M., Liu Y. X., Liu Y. D. (2024). Salvianolic Acid B Protects Against UVB-Induced Skin Aging via Activation of NRF2. *Phytomedicine*.

[32] Xu S., Sun X., Zhu Z., Xin Y., Chen C., Luo J. (2024). The Extract of Buds of *Chrysanthemum morifolium* Ramat Alleviated UVB-Induced Skin Photoaging by Regulating MAPK and Nrf2/ARE Pathways. *Journal of Ethnopharmacology*.

[33] Feng G., Chen Q., Liu J. (2024). A Non-Bactericidal Cathelicidin With Antioxidant Properties Ameliorates UVB-Induced Mouse Skin Photoaging via Intracellular ROS Scavenging and Keap1/Nrf2 Pathway Activation. *Free Radical Biology and Medicine*.

[34] Bi Y., Qiao X., Cai Z. (2025). Exosomal miR-302b Rejuvenates Aging Mice by Reversing the Proliferative Arrest of Senescent Cells. *Cell Metabolism*.

[35] Kim J. M., Kim S. Y., Noh E. M. (2018). Reversine Inhibits MMP-1 and MMP-3 Expressions by Suppressing of ROS/MAPK/AP-1 Activation in UV-Stimulated Human Keratinocytes and Dermal Fibroblasts. *Experimental Dermatology*.

[36] Luo D., Mao Y., Zhang S., Shen S., Ge X., Zhang L. (2025). Milk-Derived Exosome-Loaded SS31 as a Novel Strategy to Mitigate UV-Induced Photodamage in Skin. *Journal of Photochemistry and Photobiology B: Biology*.

[37] Sun Z., Zheng Y., Wang T. (2025). *Aloe Vera* Gel and Rind-Derived Nanoparticles Mitigate Skin Photoaging via Activation of Nrf2/ARE Pathway. *International Journal of Nanomedicine*.

[38] Nguyen D. D. N., Vu D. M., Vo N. (2024). Skin Rejuvenation and Photoaging Protection Using Adipose-Derived Stem Cell Extracellular Vesicles Loaded With Exogenous Cargos. *Skin Research and Technology*.

[39] Yan T., Huang L., Yan Y., Zhong Y., Xie H., Wang X. (2023). Bone Marrow Mesenchymal Stem Cell-Derived Exosome miR-29b-3p Alleviates UV Irradiation-Induced Photoaging in Skin Fibroblast. *Photodermatology, Photoimmunology & Photomedicine*.

[40] Gao W., Yuan L. M., Zhang Y. (2023). miR-1246-Overexpressing Exosomes Suppress UVB-Induced Photoaging via Regulation of TGF-β/Smad and Attenuation of MAPK/AP-1 Pathway. *Photochemical & Photobiological Sciences*.

[41] Wu Y., Sun K., Tu Y. (2024). miR-200a-3p Regulates Epithelial-Mesenchymal Transition and Inflammation in Chronic Rhinosinusitis With Nasal Polyps by Targeting ZEB1 via ERK/p38 Pathway. *International Forum of Allergy & Rhinology*.

[42] Gong Y., Mao J., Wu D. (2018). Circ-ZEB1.33 Promotes the Proliferation of Human HCC by Sponging miR-200a-3p and Upregulating CDK6. *Cancer Cell International*.

