# Supplementary material for: miR-200a-3p in Human Umbilical Cord Mesenchymal Stem Cell-Derived Exosomes Attenuates UVB-Induced Skin Inflammatory Response and Oxidative Stress via Keap1-Nrf2 Pathway
Source: Stem Cells Int. 2025 Oct 28;2025:7831890. doi: 10.1155/sci/7831890 (PMC12585850; doi:10.1155/sci/7831890)
Supplement: Supporting Information — Figure S1: CAT, NQO1, and SOD2 mRNA expression in HaCaTs after different treatments. Figure S2: Keap1 is predicted to be targeted by 30 miRNAs in miRDB. Figure S3: Expression levels of seven miRNAs in sequencing data. Table S1: RT-qPCR primer sequences. [file 7831890.f1.docx]

**Supplementary data**

Fig. S1


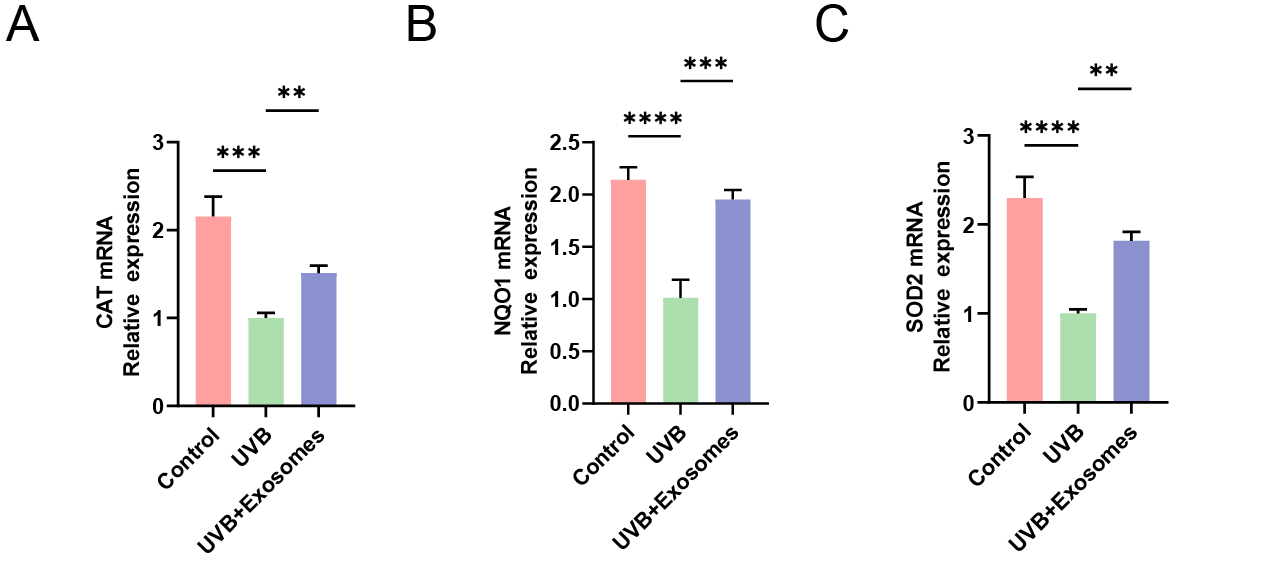


Figure S1. HuMSC-Exos reduced oxidative stress in UVB-exposed HaCaTs by modulating the Nrf2 signaling pathway. A-C. *CAT*, *NQO1* and *SOD2* mRNA expression in HaCaTs after different treatments. Data was presented as mean ± SD (n = 3). (ns: nonsignificant, *: P < 0.05, **: P < 0.01, ***: P < 0.001, ****: P < 0.0001).

Fig. S2


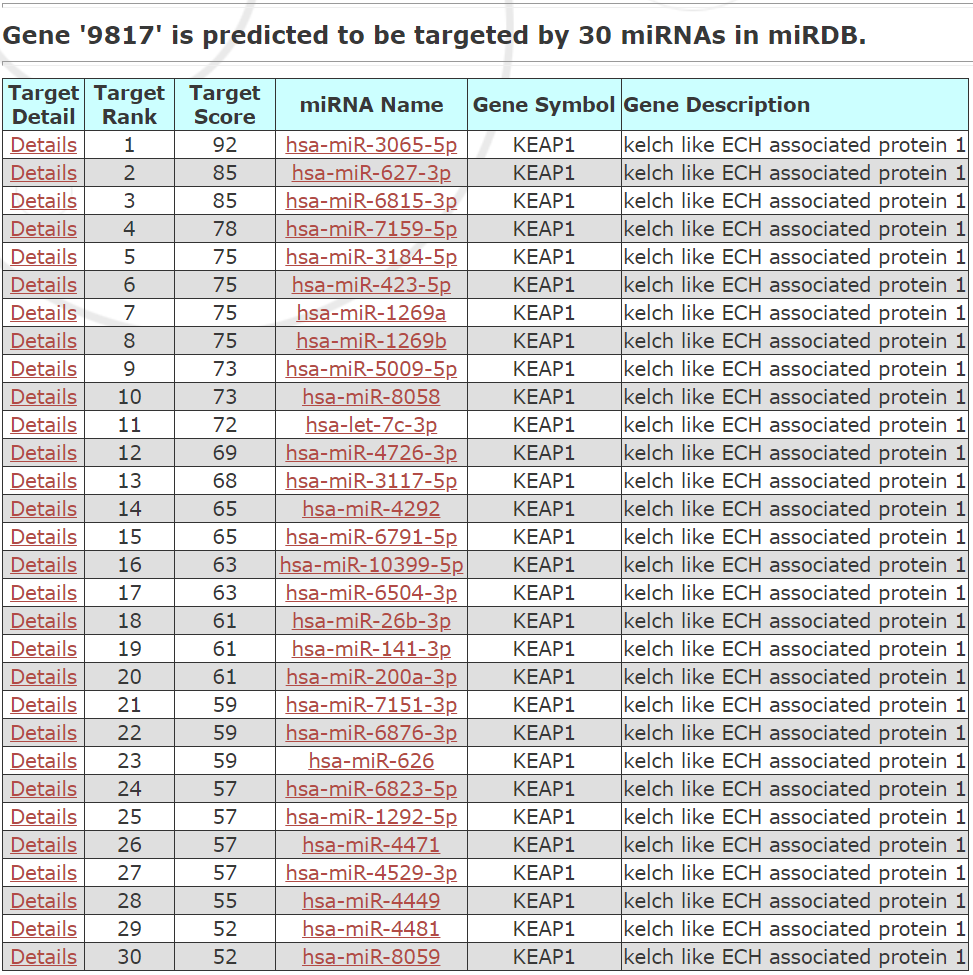


Figure S2. Keap1 is predicted to be targeted by 30 miRNAs in miRDB.

Fig. S3


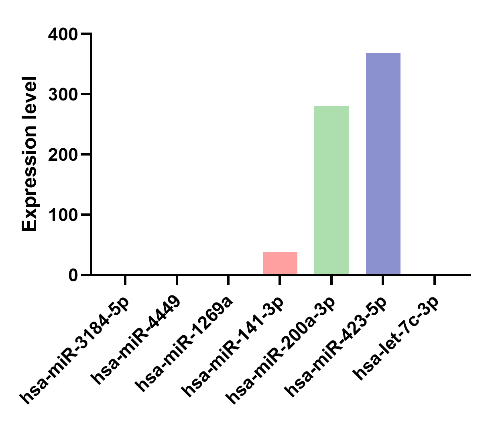


Figure S3. Expression levels of seven miRNAs in sequencing data.

**Table S1.** **Primer sequences list**

| Gene | Forward primer (5' - 3') | Reverse primer (5' - 3') |
| --- | --- | --- |
| *IL1B* Human | TTCGACACATGGGATAACGAGG | TTTTTGCTGTGAGTCCCGGAG |
| *TNF* Human | GAGGCCAAGCCCTGGTATG | CGGGCCGATTGATCTCAGC |
| *IL6* Human | ACTCACCTCTTCAGAACGAATTG | CCATCTTTGGAAGGTTCAGGTTG |
| *NOS2* Human | TTCAGTATCACAACCTCAGCAAG | TGGACCTGCAAGTTAAAATCCC |
| *PTGS2* Human | CTGGCGCTCAGCCATACAG | CGCACTTATACTGGTCAAATCCC |
| *Keap1* Human | CTGGAGGATCATACCAAGCAGG | GGATACCCTCAATGGACACCAC |
| *NFE2L2* Human | TCCAGTCAGAAACCAGTGGAT | GAATGTCTGCGCCAAAAGCTG |
| *HMOX1* Human | AAGACTGCGTTCCTGCTCAAC | AAAGCCCTACAGCAACTGTCG |
| *CAT* Human | TGGGATCTCGTTGGAAATAACAC | TCAGGACGTAGGCTCCAGAAG |
| *NQO1* Human | GAAGAGCACTGATCGTACTGGC | GGATACTGAAAGTTCGCAGGG |
| β-Actin Human | CATGTACGTTGCTATCCAGGC | CTCCTTAATGTCACGCACGAT |
| *SOD2* Human  hsa-miR-423-5p  hsa-miR-200a-3p  U6 | GCTCCGGTTTTGGGGTATCTG  GTGAGGGGCAGAGAGCGA  TAACACTGTCTGGTAACGATGT  CTCGCTTCGGCAGCACA | GCGTTGATGTGAGGTTCCAG  AGTGCAGGGTCCGAGGTATT  CATCTTACCGGACAGTGCTGGA  AACGCTTCACGAATTTGCGT |
| *Il1b* Mouse | GAAATGCCACCTTTTGACAGTG | TGGATGCTCTCATCAGGACAG |
| *Tnf* Mouse | CAGGCGGTGCCTATGTCTC | CGATCACCCCGAAGTTCAGTAG |
| *Il6* Mouse | CTGCAAGAGACTTCCATCCAG | AGTGGTATAGACAGGTCTGTTGG |
| *Cat* Mouse | GGAGGCGGGAACCCAATAG | GTGTGCCATCTCGTCAGTGAA |
| *Nqo1* Mouse | AGGATGGGAGGTACTCGAATC | TGCTAGAGATGACTCGGAAGG |
| *Sod2* Mouse | AGACCTGCCTTACGACTATGG | CTCGGTGGCGTTGAGATTGTT |
| β-Actin Mouse | GGCTGTATTCCCCTCCATCG | CCAGTTGGTAACAATGCCATGT |
| *Keap1* Mouse  *Nfe2l2* Mouse  *Hmox1* Mouse | CGGGGACGCAGTGATGTATG  TAGATGACCATGAGTCGCTTGC  AGGTACACATCCAAGCCGAGA | TGTGTAGCTGAAGGTTCGGTTA  GCCAAACTTGCTCCATGTCC  CATCACCAGCTTAAAGCCTTCT |
